# Parkinson's disease proteins: Novel mitochondrial targets for cardioprotection

**DOI:** 10.1016/j.pharmthera.2015.10.005

**Published:** 2015-12

**Authors:** Uma A. Mukherjee, Sang-Bing Ong, Sang-Ging Ong, Derek J. Hausenloy

**Affiliations:** a The Hatter Cardiovascular Institute, Institute of Cardiovascular Science, University College London, London, UK; b Cardiovascular and Metabolic Disorders Program, Duke-National University of Singapore, Singapore; c National Heart Research Institute Singapore, National Heart Centre Singapore, Singapore; d The National Institute of Health Research University College London Hospitals Biomedical Research Centre, London, UK; e Stanford Cardiovascular Institute, Stanford University School of Medicine, CA, USA

**Keywords:** CHD, Coronary heart disease, Cyp D, Cyclophilin D, Erk 1/2, Extracellular signal-regulated kinases 1/2, ETC, Electron transport chain, GPCRs, G-protein coupled receptors, IMM, Inner mitochondrial membrane, IPC, Ischemic preconditioning, IPSCs, Induced pluripotent stem cells, IPost, Ischemic postconditioning, ISCM, Ischemic cardiomyopathy, IRI, Ischemia/reperfusion injury, Mfn2, Mitofusin-2, MIBG, ^123^I-metaiodobenzylguanidine, MPP, 1-methyl-4-pyridinium, mPTP, Mitochondrial permeability transition pore, PD, Parkinson's disease, PINK1, PTEN-induced putative kinase 1, PKC, Protein kinase C, PTEN, Phosphatase and tensin homologue, RISK, Reperfusion injury salvage kinase pathway, ROS, Reactive oxygen species, VCAM-1, Vascular cell adhesion molecule 1, MEFs, mouse embryonic fibroblasts, Coronary heart disease, Parkinson’s disease, myocardial ischaemia-reperfusion injury, mitochondria, ischaemic preconditioning

## Abstract

Ischemic heart disease (IHD) is the leading cause of death and disability worldwide. Therefore, novel therapeutic targets for protecting the heart against acute ischemia/reperfusion injury (IRI) are required to attenuate cardiomyocyte death, preserve myocardial function, and prevent the onset of heart failure. In this regard, a specific group of mitochondrial proteins, which have been linked to familial forms of Parkinson's disease (PD), may provide novel therapeutic targets for cardioprotection. In dopaminergic neurons of the substantia nigra, these PD proteins, which include Parkin, PINK1, DJ-1, LRRK2, and α-synuclein, play essential roles in preventing cell death—through maintaining normal mitochondrial function, protecting against oxidative stress, mediating mitophagy, and preventing apoptosis. These rare familial forms of PD may therefore provide important insights into the pathophysiology underlying mitochondrial dysfunction and the development of PD. Interestingly, these PD proteins are also present in the heart, but their role in myocardial health and disease is not clear. In this article, we review the role of these PD proteins in the heart and explore their potential as novel mitochondrial targets for cardioprotection.

## 1 Introduction

Ischemic heart disease (IHD) is the leading cause of death and disability worldwide. The major clinical manifestations of IHD arise from the detrimental effects of acute ischemia/reperfusion injury (IRI) on the myocardium. Therefore, novel therapeutic targets for protecting the heart against IRI are required to limit cardiomyocyte death, preserve myocardial function and prevent the onset of heart failure.

In this regard, a specific group of mitochondrial proteins that have been linked to familial forms of Parkinson's disease (PD) may provide novel therapeutic targets for cardioprotection. In dopaminergic neurons of the substantia nigra, these PD proteins, which include Parkin, PINK1, DJ-1, LRRK2, and α-synuclein, have been reported to play essential roles in preventing cell death. This is achieved by maintaining normal mitochondrial function, protecting against oxidative stress, mediating mitophagy and preventing apoptosis. These rare familial PD proteins may provide important insights into the pathophysiological mechanisms underlying mitochondrial dysfunction and the development of PD. Crucially, these PD proteins are also present in the heart, but their role in myocardial health and disease is not clear. In this article, we review the role of these PD proteins in the heart and as potential therapeutic targets for cardioprotection.

## 2 Parkinson's disease

After Alzheimer's disease, PD is the second most common neurodegenerative disorder (de Lau & Breteler, 2006). The prevalence of PD in industrialized countries is estimated at 0.3% of the entire population (Nussbaum & Ellis, 2003) and approximately 1.0% of the population over the age of 60 is affected (Abou-Sleiman et al., 2006). The disease is expected to impose an increasing health, social and economic burden on societies with aging populations. Clinically, PD is characterized by rigidity, bradykinesia, resting tremor and postural instability. At the subcellular level, there is a loss of dopaminergic neurons particularly in the substantia nigra pars compacta region of the brain. Most PD cases are sporadic with only a small proportion (about 5–10%) being attributable to genetic mutations—these result in rare familial forms of PD (Mounsey & Teismann, 2011).

Interestingly, several clinical studies have reported an increased risk of cardiovascular disease in PD patients, with a lower life expectancy than the general population (Morgante et al., 2000), with heart failure (Fernandez & Lapane, 2002) and IHD (Ben-Shlomo & Marmot, 1995) being common causes of death in elderly PD patients. The prevalence of heart failure in elderly PD patients was shown in one cross-sectional study to be over twice that of age-matched non-PD patients (Zesiewicz et al., 2004). The reasons for this are unclear but may relate to several factors including the disease process itself, cardiac sympathetic denervation (Goldstein et al., 2000), PD medications (associated with heart valve disease) (Pritchett et al., 2002), and concurrent co-morbidities (such as age hypertension, diabetes, IHD and so on). Several of the mitochondrial proteins which have been linked to PD are present in the heart and are reviewed in the subsequent sections.

## 3 Parkin

### 3.1 Introduction

Parkin (PARK2) was the first gene to be associated with autosomal recessive PD (Kitada et al., 1998). It encodes a 52 kDa protein, which is found in the liver, kidney, testis, brain, skeletal muscle and heart (Kitada et al., 1998). Parkin is an E3 ubiquitin ligase which catalyzes the transfer of ubiquitin from an E2 ubiquitin-conjugating enzyme to a protein substrate in a process called ubiquitination (reviewed in Winklhofer, 2014). Under basal conditions, Parkin is located mainly in the cytosol, where its ubiquitin ligase activity is inhibited. Following cellular stress, Parkin translocates to damaged mitochondria in a PINK1-dependent manner to ubiquitinate a number of mitochondrial substrates mediating mitochondrial fragmentation, degradation and mitophagy (Matsuda et al., 2010).

#### 3.1.1 Parkin-mediated mitophagy

**Fig. 1:**
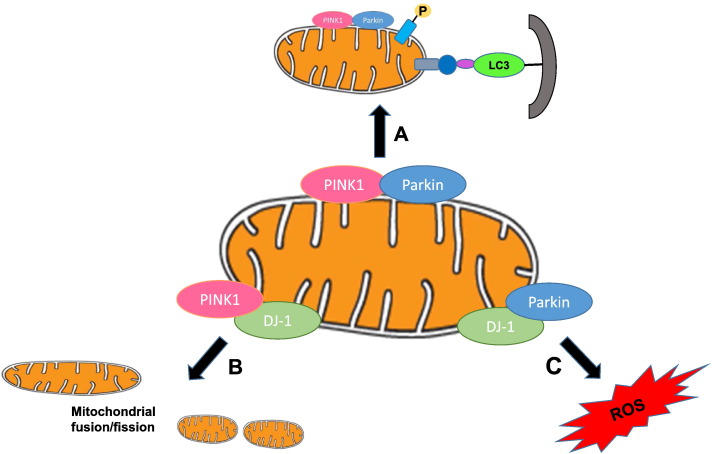
Schematic diagram showing the interplay between PINK1, Parkin, and DJ-1. (**A**) PINK1 accumulation leads to phosphorylation of mitochondrial proteins leading to recruitment of Parkin. Parkin then ubiquinates its substrates leading to the localization of P62 and LC3 for subsequent mitophagy. (**B**) PINK1 and DJ-1 are recruited to the mitochondrial outer membrane and coordinate mitochondrial dynamics in a parallel fashion. (**C**) Parkin and DJ-1 interact with each other in an oxidative stress-dependent manner.

PINK1 and Parkin have been shown to mediate the selective removal of damaged mitochondria by inducing mitophagy (reviewed in Winklhofer, 2014) (Fig. 1). In healthy mitochondria with normal membrane potential, PINK1 is imported into the mitochondria and becomes degraded by proteolysis. However, in damaged mitochondria, the depolarization of the membrane potential allows the stabilization of PINK1 in the outer mitochondrial membrane (OMM), where it phosphorylates ubiquitin resulting in the activation of Parkin (Iguchi et al., 2013, Koyano et al., 2014). The latter then ubiquitinates mitochondrial proteins (such as Mfn1 and Mfn2 and other proteins) (Gegg et al., 2010, Poole et al., 2010) resulting in mitochondrial fragmentation and the initiation of mitophagy. An additional mechanism for Parkin translocation has recently been described in which Mfn2 is phosphorylated by PINK1 and acts as a receptor for Parkin (Chen et al., 2013).

### 3.2 Parkin and the heart

Parkin is found to be expressed in the heart and recent experimental studies are beginning to elucidate its potential roles in this organ. Most of its effects in the heart appear to relate to its beneficial effects on maintaining mitochondrial health.

#### 3.2.1 Preconditioning cardioprotection

The heart can be protected against acute IRI by subjecting it to one or more episodes of brief ischemia/reperfusion—a cardioprotective phenomenon which has been termed ischemic preconditioning (IPC) and which manifests either immediately (classical IPC) or 24 hours later (delayed IPC) (Murry et al., 1986, Hausenloy, 2013, Hausenloy and Yellon, 2013). In 2011, Huang et al. (2011) were the first to investigate a role for Parkin in the heart. They found that the endogenous cardioprotective phenomenon of IPC induced the mitochondrial translocation of Parkin in *ex vivo* rat and *in vivo* murine hearts (Huang et al., 2011). Crucially, Parkin-deficient mice were resistant to IPC, suggesting that Parkin is required to mediate the cardioprotection elicited by IPC (Huang et al., 2011). Kubli et al. (2013) showed that cardiac size and function were normal under baseline conditions in Parkin-deficient mice when compared to wild-type mice. However, Parkin-deficient hearts were more susceptible to ischemic injury following permanent left coronary artery occlusion as evidenced by larger infarct sizes, worse left ventricular remodeling and increased mortality (Kubli et al., 2013). Together, these findings suggest that Parkin might play a role in maintaining mitochondrial function under stress conditions; while the role of Parkin as a mediator of IPC is not clear, it may be related to the former's role in mediating mitophagy.

#### 3.2.2 Mitophagy

A Parkin-independent mitophagy pathway involving Dynamin-related protein 1 (Drp1) has recently been described (Kageyama et al., 2014), which may in part explain the lack of cardiac phenotype in Parkin-deficient hearts in the study by Kubli and colleagues (Kubli et al., 2013). A separate study by Song et al. (2015) reported that endogenous Parkin is rare in normal hearts but upregulated in response to Drp1 ablation leading to a cardiomyopathy phenotype. Interestingly, concomitant deletion of both Parkin and Drp1 reversed this phenotype demonstrating the complex interplay between Parkin and mitochondria. Hoshino et al. (2013) have investigated the role of Parkin-mediated mitophagy in the aged heart and in the heart subjected to doxycycline cardiotoxicity. In both these settings, they found that cytosolic p53 prevented the mitochondrial translocation of Parkin thereby attenuating the protective mitophagic response (Hoshino et al., 2013).

#### 3.2.3 Mitochondrial dynamics

Mitochondrial dynamics have also been implicated in cardiomyopathy secondary to impaired mitophagy. Parkin-knockout Drosophila displayed enlarged hollow “donut” mitochondria, which could be rescued by cardiomyocyte-specific Parkin expression and by also suppressing cardiomyocyte mitochondrial fusion. The latter also reversed the dilated cardiomyopathy phenotype. The group thereby inferred that Parkin ablation supressed normal mitophagic organelle elimination; this resulted in a contamination of abnormal mitochondria, which were able to fuse with normal mitochondria to precipitate cardiomyopathy. By suppressing mitochondrial fusion factors and therefore interrupting the feed-forward pathway of mitochondrial contamination, Bhandari et al. prevented this cardiomyocyte mitochondrial pool contamination and found that end-organ dysfunction could potentially be delayed (Bhandari et al., 2014). Although mitochondrial morphology by electron microscopy revealed disorganized clusters of small mitochondria, there was no difference in mitochondrial respiratory function and mitochondrial permeability transition pore (MPTP) opening susceptibility (Kubli et al., 2013).

## 4 PINK1

### 4.1 Introduction

Mutations in PTEN-induced kinase 1—PINK1 (PARK6), a mitochondrially targeted serine/threonine kinase, are the second most frequent cause of autosomal recessive, young-onset PD after Parkin (PARK2) (Abramov et al., 2011). The protein is expressed in the brain, heart, skeletal muscle, liver, kidney, pancreas and testes. PINK1 is a mitochondrial protein whose kinase domain localizes in the OMM. Its kinase activity is critical for its protective effects—indeed, most mutations are found in the serine/threonine kinase domain, which suggests that loss of kinase activity plays a crucial part in the pathogenesis of PINK1-linked PD (Zhou et al., 2008, Hatano et al., 2009). PINK1 has been identified as a protective agent against oxidative stress-induced apoptosis—one of the mechanisms underlying this is through its phosphorylation of the mitochondrial chaperone TNF-receptor-associated protein 1, (TRAP1) (Pridgeon et al., 2007).

Experiments have shown that the expression of PINK1 *in vitro* protects against exogenous stressors such as MPP+, rotenone and staurosporine (Petit et al., 2005, Haque et al., 2008, Sandebring et al., 2009). Conversely, siRNA ablation of PINK1 enhanced cell death in the presence of certain toxins. Fibroblasts from patients with mutations in PINK1 displayed significant bioenergetics defects, such as reduced mitochondrial membrane potential, altered redox state, and enhanced sensitivity to calcium stimulation. Similarly, mitochondria isolated from PINK1-KO flies have been shown to have reduced respiratory function (Liu et al., 2011).

Mitochondrial morphology is also impaired when PINK1 is suppressed—there is an increase in fragmented mitochondria in PINK1 knockdown mammalian cultured cells (Deng et al., 2005, Exner et al., 2007, Lutz et al., 2009). Additionally, transgenic Drosophila with PINK1 promotes mitochondrial fission in dopaminergic neurons, while complete ablation of PINK1 leads to excessive fusion (Yang et al., 2008).

### 4.2 PINK1 and the heart

Siddall et al. (2008) were the first to postulate a cardioprotective role for PINK1 in the heart. Since then, Billia et al. (2011) have demonstrated that levels of PINK1 were significantly reduced in end-stage human heart failure and that PINK1 activity is necessary for correct postnatal myocardial development. The authors found that PINK1-deficient mice had mitochondrial impairment; in particular, they were more sensitive toward Reactive oxygen species (ROS)-dependent depolarization of the mitochondrial membrane potential, had decreased oxidative phosphorylation and more mitochondrial swelling. Our grouphave confirmed similar mitochondrial findings in isolated cardiomyocytes following simulated IRI (Siddall et al., 2013). Furthermore, we showed that mice genetically ablated for PINK1 developed significantly larger myocardial infarcts following an episode of sustained 35 minutes of regional ischemia and 30 minutes of reperfusion compared to their wild-type counterparts, while mice heterozygous for PINK1 had intermediate infarct sizes (Siddall et al., 2013).

Billia et al. (2011) also described greater oxidative stress and higher levels of lipid peroxidation in PINK1-KO mice. Recent work has centered on cardiolipin, a mitochondrial phospholipid that is crucial to maintaining normal cardiac function. It had been found that ALCAT1, a lysocardiolipin acyltransferase that catalyzes pathological remodeling of cardiolipin in response to oxidative stress in cardiomyopathy, leads to ROS production and mitochondrial dysfunction (Li et al., 2010). Ablation of ALCAT1 dramatically increased expression of PINK1, which supports a protective function of PINK1 against oxidative stress and cardiomyopathy (Liu et al., 2012).

## 5 DJ-1

### 5.1 Introduction

In 1997, Nagakubo et al. (1997) first identified DJ-1 to be a novel oncogene capable of transforming mouse NIH3T3 cells in cooperation with activated *ras*. The human DJ-1 protein has 189 amino acids (20 kDa protein) and belongs to the ThiJ/PfpI family where it functions as a dimer (Tao & Tong, 2003). It has been shown to be present in a number of human tissues including the brain, liver, skeletal muscle, kidney, testes, as well as the heart (Nagakubo et al., 1997). In 2003, it was discovered that deletion and point mutations in the DJ-1 gene on chromosome 1p36 (Leu^166^Pro or L166P) were responsible for the PARK7 familial form of PD (Van Duijn et al., 2001, Bonifati et al., 2003). The L166P mutant form of DJ-1 disrupts the C-terminal region, which is required for its dimerization and subsequent activation (Olzmann et al., 2004), thereby increasing its degradation (Hulleman et al., 2007).

The first study to implicate DJ-1 as a neuroprotective protein was by Yokota and co-workers in 2003 (Yokota et al., 2003), who demonstrated that ablating DJ-1 increased cell death induced by oxidative stress, endoplasmic reticulum stress and proteosome inhibition, whereas overexpressing wild-type DJ-1 but not the L166P DJ-1 mutant protected against cell death in mouse Neuro2a cells and human embryonic kidney 293T cells. The neuroprotective effect which is absent in the L166P DJ-1 mutant has been confirmed by a number of subsequent studies in response to oxidative stress (Miller et al., 2003, Moore et al., 2003, Takahashi-Niki et al., 2004, Ren et al., 2012, Chang et al., 2014), proteosome inhibition (Moore et al., 2003) and apoptotic stimuli (Moore et al., 2003, Ren et al., 2012). Neurons in mice lacking DJ-1 were also demonstrated to be more susceptible to oxidative stress (Kim et al., 2005b). Injection of wild-type DJ-1 into murine brains has been reported to reduce cerebral infarct size in both ischemic and endothelin-1-induced murine models of stroke (Aleyasin et al., 2007, Yanagisawa et al., 2008). The role that DJ-1 plays in the pathophysiology of PD remains unclear, but the beneficial effects of this protein in terms of mediating neuroprotection are summarized below:

• *Anti-oxidant properties*: Oxidative stress is known to be a critical determinant in the pathogenesis of PD. In 2001, Mitsumoto et al. (Mitsumoto & Nakagawa, 2001) first demonstrated that DJ-1 may act as an endogenous indicator of oxidative stress, suggesting that it may play a role as an anti-oxidant. Subsequent studies have demonstrated that the Cys-106 residue on the DJ-1 protein is the site of oxidation, resulting in the formation of cysteine-sulphinic acid, a process which appears critical to its mitochondrial translocation and neuroprotective effect (Canet-Avilés et al., 2004) (Fig. 1).
• *Anti-apoptotic effects*: DJ-1 has been reported to be present in the nucleus and can modulate transcription (Nagakubo et al., 1997, Niki et al., 2003, Junn et al., 2005, Junn et al., 2009). In 2005, it was proposed by Junn and co-workers (Junn et al., 2005) that DJ-1 reduced cell death by interacting with and sequestering the death-associated protein, Daxx, in the nucleus, thereby preventing its cytosolic translocation and activation of the apoptosis signal-regulating kinase 1 (ASK1), a pro-apoptotic protein which activates JNK1/2. More recent studies have suggested that DJ-1 may inhibit oxidative stress-induced apoptotic cell death by interacting with ASK1 directly (Im et al., 2010, Mo et al., 2010, Klawitter et al., 2013) and preventing its dissociation with Thioredoxin 1, a factor which inhibits ASK1 activity under basal conditions and reducing JNK activity (Im et al., 2010). Furthermore, DJ-1 has been reported to prevent apoptotic cell death by activating and phosphorylating the anti-apoptotic protein kinase, Akt, by suppressing PTEN activity (Kim et al., 2005a, Aleyasin et al., 2010). Conversely, DJ-1 ablation resulted in down-regulation of the PI3K-Akt pathway and cell death (Kim et al., 2005a, Aleyasin et al., 2010). Recent studies suggest that DJ-1 may actually stabilize hypoxia-inducible factor through its activation of the PI3K-Akt pathway (Vasseur et al., 2009).
• *Effects on mitochondrial respiratory function*: Impaired mitochondrial respiration arising from complex I inhibition has been implicated as a causative factor in mitochondrial dysfunction and the production of oxidative stress observed in PD. Under basal conditions, endogenous DJ-1 is present in the cytosol and nucleus (Canet-Avilés et al., 2004, Taira et al., 2004, Zhang et al., 2005). Upon oxidation, DJ-1 translocates from the cytosol to the OMM, a process which is required for its neuroprotective effect (Junn et al., 2009). Interestingly, a more detailed study has suggested that endogenous DJ-1 is present in the mitochondrial inter-membranous space and matrix of neuronal cells (Zhang et al., 2005). It has been suggested that DJ-1 binds to electron transport chain complexes (NDUFA4 and ND1) and is required for the latter's normal function such that knockdown of DJ-1 inhibits complex I activity (Hayashi et al., 2009). Experimental studies have demonstrated that overexpressing wild-type DJ-1 protected against rotenone-induced (a complex I inhibitor) induced mitochondrial dysfunction and that the ablation of endogenous DJ-1 increased the susceptibility of neurons to rotenone and other complex I inhibitors (Mullett and Hinkle, 2009, Thomas et al., 2011, Gao et al., 2012). A recent study has also shown that mouse embryonic (MEFs) lacking DJ-1 had impaired mitochondrial respiration due to complex I inhibition, increased mitochondrial oxidative stress, reduced mitochondrial membrane potential, more fragmented mitochondria, and impaired mitophagy, findings which confirmed that DJ-1 is required for normal mitochondrial function (Krebiehl et al., 2010). The beneficial effect of DJ-1 on mitochondrial function and autophagy appears to be parallel to the PINK-1/Parkin pathway, two other proteins in which mutations are associated with Parkinson's disease (Thomas et al., 2011; reviewed in Winklhofer and Haass, 2010, Chu, 2011).
• *Effects on mitochondrial morphology*: Impaired mitochondrial DJ-1 deficiency leads to altered mitochondrial morphology and increases the production of ROS as a result of altered mitochondrial dynamics (Irrcher et al., 2010). DJ-1 has also been shown to promote endoplasmic reticulum (ER)-mitochondria tethering in HeLa cells, albeit mitochondrial elongation was actually induced (Ottolini et al., 2013). Similar findings were also observed in neuronal cells (Wang et al., 2012). Overexpression of DJ-1 in the HL-1 cardiac cell line induces mitochondrial elongation and decreases sensitivity to mPTP opening. Although mice deficient in DJ-1 have more fragmented mitochondria, the hearts of these mice do not display any apparent cardiac phenotype (Dongworth et al., 2014). Nevertheless, the effect of DJ-1 on mitochondrial morphology seems to be species and cell-type dependent (Hao et al., 2010).
• *Effects on mitochondrial turnover*: The regulation of mitochondrial turnover is vital for cellular homeostasis and survival (Farré et al., 2009) and DJ-1 may also be involved in the quality control of mitochondria; there is dysfunctional lysosomal degradation and reduced mitochondrial clearance in DJ-1 KO MEFs (Krebiehl et al., 2010). Autophagy is a lysosomal degradation process that maintains sufficient ATP levels during periods of energy limitation (Vasseur et al., 2009) and plays a protective role in the early stages of apoptosis as part of the cellular attempt at self-rescue (Jellinger and Stadelmann, 2000, González-Polo et al., 2007a, González-Polo et al., 2007b). There is reduced basal autophagy encountered in DJ-1 −/− MEFs cells (Krebiehl et al., 2010).
• *Effects on mitochondrial biogenesis*: Impaired mitochondrial DJ-1 also promotes the transcriptional activity of peroxisome proliferator-activated receptor-γ co-activator 1α (PGC-1α), an agent involved in mitochondrial biogenesis and the oxidative stress response (Zhong & Xu, 2008). DJ-1 inhibits the SUMOylation of a transcriptional repressor of PGC-1α, pyrimidine tract-binding protein-associated splicing factor (PSF) and, in so doing, raises levels of manganese superoxide dismutase, which aids in oxidative stress defence (Zhong & Xu, 2008).
• *Interactions with other Parkinson's proteins*: DJ-1-dependent mitochondrial defects can be rescued by addition of a cell-permeable glutathione precursor or Parkin/PINK1 overexpression (Thomas et al., 2011). Recently, DJ-1 was found to negatively regulate PINK1-dependent Parkin translocation to depolarized mitochondria in neurons, as a result of its ability to control Reactive oxygen species (ROS) generation (Joselin et al., 2012). DJ-1 can act on mitochondria to prevent the aggregation and toxicity of α-synuclein in an oxidation-dependent manner (Batelli et al., 2008, Giaime et al., 2012). Parkin and DJ-1 may interact under conditions of oxidative stress (Moore et al., 2005). In cells overexpressing PINK1, DJ-1 binds to PINK1 and increases its steady-state levels (Tang et al., 2006). PINK1 and Parkin have also been found to rescue the phenotype of DJ-1 −/− cells (Irrcher et al., 2010) (Fig. 1).

### 5.2 DJ-1 and the heart

DJ-1 is known to be present in the heart and only recently has its role in this tissue been investigated. Under baseline conditions, mice lacking DJ-1 do not appear to have an obvious cardiac phenotype in terms of heart/body weight ratios, cardiomyocyte cross-section, cardiac size and function on echocardiography when compared to wild-type mice, suggesting a non-essential role of this mitochondrial protein under physiological conditions (Billia et al., 2013, Dongworth et al., 2014). However, under conditions of cellular stress, hearts deficient in DJ-1 appear to be more susceptible to a number of pathological stresses. Neonatal murine cardiomyocytes overexpressing DJ-1 have been shown to be protected against cell death induced by oxidative stress, whereas cardiomyocytes deficient in DJ-1 were more susceptible to ROS-induced apoptotic cell death (Billia et al., 2013).

#### 5.2.1 Ischemia/reperfusion injury

A number of recent experimental studies have suggested that DJ-1 protects the heart against acute IRI (Bitar et al., 2012, Liu et al., 2014). Proteomic analysis of murine hearts subjected to *in vivo* acute IRI demonstrated that protein levels of DJ-1 were down-regulated after 30 minutes of ischemia, a change which persisted 60 minutes into reperfusion but which was restored by 120 minutes of reperfusion. These changes in myocardial DJ-1 protein expression were not associated with changes in mRNA expression of DJ-1 implicating degradation in DJ-1 protein levels in response to acute IRI. It has been shown that DJ-1 becomes unstable and is degraded in response to oxidative stress, a process which is exacerbated by the down-regulation of the antioxidant transcriptional master regulator Nrf2—whether this is the mechanism for DJ-1 down-regulation with IRI remains to be determined (Bitar et al., 2012, Liu et al., 2014).

Overexpression of DJ-1 in H9c2 myotubes and the HL-1 cardiomyocytes has been shown to protect cells against simulated ischemia (Yu et al., 2013) and simulated IRI (Dongworth et al., 2014). The protective effects of DJ-1 were associated with inhibition of MPTP opening and increased mitochondrial elongation in HL-1 cardiomyocytes, markers of cardioprotection (Dongworth et al., 2014). Importantly, cells overexpressing non-functional mutant forms of DJ-1 (L166P and Cys106A) were not protected against simulated IRI (Dongworth et al., 2014).

The role of endogenous DJ-1 in the setting of acute IRI has also been recently examined. Billia et al. (2013) reported that DJ-1-deficient mice had larger infarct sizes and greater levels of interstitial fibrosis 4 weeks following an episode of acute IRI; these findings were associated with a significant increase in the number of apoptotic cardiomyocytes in the peri-infarct area 72 hours following IRI when compared with wild-type animals. Dongworth et al. (2014) also reported an acute increase in MI size in DJ-1-deficient mice subjected to 45 minutes of myocardial ischemia and 24 hours of reperfusion, findings which were associated with mitochondrial fragmentation on electron microscopy, but no differences in calcium-induced MPTP opening, mitochondrial respiratory function, or myocardial ATP levels, when compared to wild-type mice. In summary, these findings suggest that DJ-1 is regulated in the setting of acute IRI and it may protect the heart against IRI and therefore be a novel target for cardioprotection.

The diabetic heart is more susceptible to acute IRI and cardiac failure. Liu et al. (2013) have investigated the role of DJ-1 in the diabetic heart in the setting of Ischemic Postconditioning (IPost) the cardioprotection induced by interrupting myocardial reperfusion with short-lived episodes of ischemia) (Zhao et al., 2003, Hausenloy, 2013). They found that protein levels of DJ-1 were reduced in the diabetic rat heart (attributed to increased myocardial oxidative stress) and these findings were associated with a blunted response to the MI-limiting effects of IPost (Liu et al., 2013).

#### 5.2.2 Cardioprotection

The role of DJ-1 as a potential mediator of IPC has been investigated in recently published experimental studies. Lu et al. (2012) found that subjecting rat heart-derived H9c2 cells to a standard hypoxic preconditioning stimulus resulted, 24 hours later, in the up-regulation of both ERK1/2 (a known mediator of delayed IPC and DJ-1, as well as lower levels of oxidative stress and cell death. Importantly, inhibition of Erk1/2 prevented the up-regulation of DJ-1 and the ablation of DJ-1 abrogated the protection associated with preconditioning, suggesting that hypoxic preconditioning mediates its protective effect through the Erk1/2-dependent activation of DJ-1 (Lu et al., 2012). We have found that mice lacking DJ-1 were resistant to the infarct-limiting effects of classical IPC, suggesting that DJ-1 may also act as a mediator of classical IPC in an *in vivo* murine MI model (Dongworth et al., 2014). The mechanism through which DJ-1 mediates the cardioprotection elicited by classical IPC is unclear and remains to be investigated. The role of DJ-1 in IPost, has been investigated by Liu et al. (2013) who found that a standard IPost protocol was able to increase DJ-1 protein levels in the non-diabetic rat heart but not diabetic ones due to impaired signaling through the PI3K-Akt pathway and increased oxidative stress.

#### 5.2.3 Pressure-overload left ventricular hypertrophy

The role of DJ-1 has been investigated in the setting of left ventricular hypertrophy (Billia et al., 2013). Mice deficient in DJ-1 have been demonstrated to be more susceptible to pressure-overload left ventricular hypertrophy induced by both total aortic constriction and Angiotensin II (Billia et al., 2013). This finding was associated with impaired cardiac contractile function, increased interstitial fibrosis, upregulated mRNA expression of known pathological cardiac proteins (such as atrial natriuretic factor and brain natriuretic peptide), Erk1/2 activation, β-myosin heavy chain, oxidative DNA damage, impaired mitochondrial function, and less angiogenesis (with reduced mRNA expression of VEGF and angiopoietin) (Billia et al., 2013).

#### 5.2.4 Heart failure

The role of DJ-1 in the development of heart failure has been recently investigated—oxidative stress is a known mediator of heart failure and DJ-1 has been demonstrated to have an anti-oxidant effect. Furthermore, Billia et al. (2013) demonstrated that protein levels of DJ-1 in left ventricular tissue were down-regulated in patients with end-stage human heart failure compared to normal healthy controls. The reduction in DJ-1 protein levels was likely to be due to post-translational modification as there was no difference in mRNA expression between the two groups (Billia et al., 2013). Interestingly, Klawitter et al. (2013) found that protein levels of DJ-1 were elevated in myocardial tissue from patients with end-stage ischemic heart failure compared to patients with end-stage dilated cardiomyopathy and control patients; this was associated with reductions in PTEN and up-regulation of Akt (Klawitter et al., 2013). The reason for this difference in DJ-1 protein expression is not clear but was attributed to a cellular response to stress (Klawitter et al., 2013).

## 6 LRRK2

### 6.1 Introduction

LRRK2 is a multi-domain protein that harbors a kinase and a GTPase domain in multi-vesicular bodies and in autophagic vesicles (Korr et al., 2006). LRRK2 resides mainly in the cytoplasm and is partially localized to the mitochondria. It is associated with membranes, such as mitochondria, ER and synaptic vesicles (Vitte et al., 2010). The G2019S mutation is the most common genetic cause of PD. Autosomal dominant mutations in LRRK2 are associated with both familial and late-onset PD and patients have clinical symptoms and pathology typical of sporadic PD (Mortiboys et al., 2010).

#### 6.1.1 Effects on mitochondria

Patients with the G2019S mutation in LRRK2 have reduced mitochondrial membrane potential and decreased total intracellular ATP levels (Mortiboys et al., 2010). The expression of the G2019S mutation in fibroblast and neuroblastoma cells in mouse brain was associated with mitochondrial uncoupling, decreased mitochondrial membrane potential and increased oxygen utilization both basally and under oligomycin-inhibited conditions. These findings are also consistent with defective oxidative phosphorylation resulting in decreased cellular ATP levels demonstrated by Mortiboys et al. (2010). The decreased mitochondrial membrane potential and normal maximal oxygen utilization rates implied an increased permeability of the inner mitochondrial membrane (IMM) to protons; however, the mPTP was not found to be involved as suggested by lack of increased ROS or death *in vitro* (Papkovskaia et al., 2012).

### 6.2 LRRK2 and the heart

LRRK2 mRNA has been detected in the heart at lower levels than in the brain (Zimprich et al., 2004). Interestingly, a recent study showed that there were no differences in the hearts of LRRK2 wild-type and knockout mice in terms of histopathology and ultrastructure (Baptista et al., 2013). A case study has recently reported a patient with LRRK2 mutation displaying features of cardiac sympathetic denervation, baroreflex-sympathoneural and baroreflex-cardiovagal failure (Goldstein et al., 2007, Gehrke et al., 2010). Unlike in idiopathic PD, heart rate variability was not reduced in LRRK2-PD; this implies a relative sparing of pathologic involvement of the autonomic innervation of the heart (Joers & Emborg, 2014).

## 7 α-synuclein

### 7.1 Introduction

This is a small, acidic protein composed of 140 amino acid residues. It is a soluble monomeric protein, with a predominantly cytosolic location, although a fraction has been identified in the mitochondria (Cole et al., 2008). Point mutations in the α-synuclein gene (PARK1) (Polymeropoulos, 1997) have been described as causes of familial PD; allele triplication of wild-type α-synuclein gene mutation leads to autosomal dominant PD (Singleton et al., 2004).

#### 7.1.1 Effects on mitochondria

• *Mitochondrial bioenergetics*: Abnormal mitochondria are found in transgenic mice overexpressing mutant α-synuclein (Song et al., 2004). It is targeted to the IMM where it binds mitochondrial complex 1 and impairs its function (Liu et al., 2009). One study found that *in vitro* incubation of rat brain mitochondria with recombinant human α-synuclein led to dose-dependent loss of mitochondrial transmembrane potential without an effect on the activities of the respiratory chain complex. This suggested that α-synuclein may impair mitochondrial bioenergetics through a direct effect on mitochondrial membranes. (Banerjee et al., 2010). In transgenic animals overexpressing A53T of α-synuclein specifically within dopaminergic neurons, the protein localized to the mitochondria as monomers and oligomers (Chinta et al., 2010), where the oligomers led to a selective inhibition of complex I function and also enhanced mitophagy under stress conditions.
• *Mitochondrial dynamics*: α-synuclein has been shown to interfere with mitochondrial dynamics by disturbance of mitochondrial fusion, with its overexpression in cells leading to mitochondrial fragmentation (Kamp et al., 2010, Nakamura et al., 2011). Nevertheless, there have also been other studies citing different effects of α-synuclein in regulating mitochondrial morphology, for instance no effects observed under physiological conditions (Zhu et al., 2012), age-dependent regulation of mitochondrial dynamics (Xie & Chung, 2012), or promoting fusion (Guardia-Laguarta et al., 2014).
• *Axonal transport of mitochondria*: α-synuclein is also thought to disrupt the transport of mitochondria by affecting the polymerization and depolymerization of tubulin, which mediate the distribution of mitochondria within the neuron (Zhou et al., 2010). While the exact mechanism through which it achieves this is unknown, it is believed that the two proteins interact in some way. Disruption of the microtubule network could significantly impact the mitochondrial dynamics and the mitophagy of damaged mitochondria (Protter et al., 2012).
• *ER stress*: ER stress has been detected in dopaminergic neurons of the substantia nigra bearing α-synuclein inclusions in the brains of patients affected by PD, indicating that ER stress is involved in sporadic PD (Jiang et al., 2010). Furthermore, the ER stress observed closely correlated with the accumulation and aggregation of α-synuclein (Jiang et al., 2010). Elevated α-synuclein levels have been found to block ER to Golgi membrane trafficking and cause ER stress (Cooper et al., 2006, Thayanidhi et al., 2010), raising the possibility that α-synuclein-induced ER stress might play a role in precipitating mitochondrial stress.

### 7.2 α-synuclein and the heart

Autonomic dysfunction may be a feature of PD and symptoms of this include chronic constipation, urinary urgency and incontinence (Niimi et al., 1999). One study investigating a family, the “Iowa kindred” who have AD parkinsonism with a triplication of the normal (not mutant) α-synuclein gene (Singleton et al., 2003) found that cardiac sympathetic denervation co-segregated with parkinsonism in this kindred. The overexpression of the normal α-synuclein resulted in both loss of dopamine-producing cells in the nigro-striatal system in the brain as well as loss of norepinephrine-producing cells in the sympathetic cardiac system (Singleton et al., 2004). Recently, Navarro-Otano and colleagues have shown that abnormal α-synuclein aggregates are present in neurons and nerve fibers (i.e. peripheral autonomic nervous system) of epicardial tissue samples obtained during cardiac surgery in over 90 subjects without parkinsonism (Navarro-Otano et al., 2013). The authors speculated that this could be a sign of early cardiac involvement in pre-motor PD.

## 8 Mitochondrial Parkinson's proteins as therapeutic targets

Evidence is emerging that some familial Parkinson's proteins play a protective role in the heart (see Table 1 and Fig. 1). It is imperative that mechanisms are sought to explain the associations between PD and cardiovascular disease, in order to better develop therapeutics to target the respective diseases. Small molecule modulators of such proteins could potentially enhance the pro-survival pathways within the heart, in which several of the proteins are involved (Zhou et al., 2011).

A recent study has identified several DJ-1-binding small molecule compounds which bind to the Cys-106 region within DJ-1 maintain activation of DJ-1 protein, thereby protecting cells against oxidative stress-induced death (Miyazaki et al., 2008). This approach is already being pursued in the field of neurodegeneration; following 48 hours of treatment with the histone deacetylase inhibitor, 4-sodium phenylbutyrate in the N27 dopamine cell line, levels of DJ-1 were increased by 300% and cells were rescued from oxidative stress (Zhou et al., 2011). Another study reported that the intrastriatal pre-injection of a DJ-1 modulator, UCP0054278, inhibited neurodegeneration arising from 90 minutes of middle cerebral artery occlusion and reperfusion in rats (Yanagida et al., 2009). Similar efforts should be applied toward developing and testing such compounds in the heart to investigate possible cardioprotective benefits.

The ability to generate patient-specific induced pluripotent stem cells (iPSCs) (Takahashi & Yamanaka, 2006) is also extremely exciting for the development and evaluation of novel therapeutics. Human iPSCs present an unprecedented opportunity to study disease-specific differences at a personalized basis and can be genetically engineered to carry mutations of specific Parkinson proteins. These iPSCs can then be differentiated into desired lineages including cardiomyocytes and subsequently used for drug screening, which takes into account individual drug responses within a patient population. The validity of this approach has been exemplified by the successful application of human iPSCs in various diseases (Itzhaki et al., 2011, Yazawa et al., 2011, Sun et al., 2012).

## 9 Summary and conclusions

**Fig. 2:**
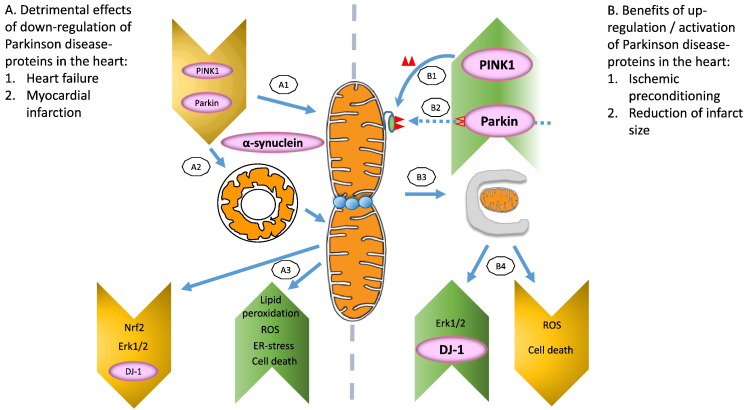
This schematic diagram illustrates the effects of Parkinson's disease-related proteins in the heart. On the left panel (**A**), (A1) down-regulation of PINK1 and Parkin, or up-regulation of α-synuclein in the inner mitochondrial membrane (IMM) leads to excessive mitochondrial fragmentation. (A2) Ablation of Parkin also promotes formation of donut-shaped mitochondria which will eventually fragment should the stress conditions be prolonged. (A3) The fragmented mitochondria have decreased levels of Nrf2, Erk1/2, and DJ-1 while the levels of lipid peroxidation, ROS, ER-stress, and cell death increase. Conversely, in the right panel (**B**), up-regulation / activation of Parkinson disease proteins in the heart belies the beneficial effects of mitophagy in ischemic pre-conditioning (IPC) and reduction of infarct size. (B1) PINK1 phosphorylates Mfn2 in the outer mitochondrial membrane (OMM) thus (B2) recruiting Parkin for mediation of mitochondrial fragmentation and (B3) mitophagy. (B4) Increased PINK1/Parkin also promotes up-regulation of Erk1/2 and DJ-1 with decreased ROS and cell death.

Given that aging is a significant contributor to both Parkinson's disease and cardiac disease, the occurrence of both these conditions is set to rise. Recent data suggest that the mitochondrial Parkinson's proteins Parkin, PINK1, DJ-1, LRRK2 and α-synuclein are present in the heart and studies are beginning to elucidate their role (see Table 1 and Fig. 2 for summary). There is emerging evidence that PINK1 and DJ-1 may act as potential mediators of cardioprotection; the mechanisms underlying this protective effect are not clear but may relate to their beneficial effects on mitochondrial function. They therefore represent potential therapeutic targets for treating patients with IHD. These PD proteins also appear to have beneficial effects in other cardiac diseases such as left ventricular hypertrophy and heart failure but further studies are required to identify the underlying mechanisms.

## Conflict of Interest Statement

The authors declare that there are no conflicts of interest.

## Figures and Tables

**Table 1:** Potential involvement of Parkinson-disease proteins in various cardiac conditions.

Parkinson-disease protein	Cardiac condition
Parkin	Acute ischemia/reperfusion injury
PINK1	Acute ischemia/reperfusion injury Heart failure, cardiac development
DJ-1	Acute ischemia/reperfusion injury Left ventricular hypertrophy, heart failure, diabetic heart
LRRK2	Heart failure?
α-synuclein	Heart failure?
